# Role of autoimmunity in hepatitis c VIRUS infection: a case report and a brief review of literature

**DOI:** 10.1186/1939-4551-8-S1-A175

**Published:** 2015-04-08

**Authors:** Fabiana Mascarenhas, Myrthes Toledo Barros, Leonardo Mendonça, Pablo Torres, Karla Boufleur, Jorge Kalil, Eduardo Longen

**Affiliations:** 1 Hcfmusp, Brazil; 2 Hospital Das Clínicas -Faculdade De Medicina –USP, Brazil; 3 University of São Paulo, Brazil

## Background

Hepatitis C virus (HCV) is an important public health problem worldwide. Immunological complications are found in 40-74% of patients with HCV, as well as the prevalence of HCV infection is higher among individuals with these conditions, suggesting a pathogenetic virus influence.

As a systemic pathology, autoimmunity may occur even without hepatic manifestations. Studies indicate that peripheral nervous system disorders have been observed as complications of HCV infection. This case report is about a 56 year old male patient with skin scaly erythematous lesions and stellate hypo pigmented spots, muscle atrophy of the extremities and peripheral neuropathy march with diffuse muscle weakness associated with a positive history of hepatitis.

## Methods

Clinical, image and laboratoy records of Hospital das Clínicas da FMUSP review. Skin biopsy, neurologyc investigation and hepatits C treatment were performed. Understanding the association between HCV and autoimmune manifestations, may light to virus testing, beyond to indicate antiviral e immunomodulatory treatment.

## Results

Biopsy of skin lesions showed inflammatory features, some with fibrinoid necrosis. Electromyography demonstrated myopathy and neuropathy pattern. Nerve biopsy revealed vasculitic neuropathy. The patient was submitted to hepatitis C treatment with purpose to control autoimmune manifestations, but had several collateral effects that required avoidance of the drug. It was also tried several immunosuppressive medications, but all tended to fail and partially control was acquired with cyclosporine.

## Conclusions

Imunossupressive treatment and hepatitis C treatment should be used to control autoimmune manifestations related to virus C infection.

## Consent

Written informed consent was obtained from the patient for publication of this abstract and any accompanying images. A copy of the written consent is available for review by the Editor of this journal.

